# The End-Diastolic Velocity of Thyroid Arteries Is Strongly Correlated with the Peak Systolic Velocity and Gland Volume in Patients with Autoimmune Thyroiditis

**DOI:** 10.1155/2017/1924974

**Published:** 2017-09-14

**Authors:** Danilo Bianchini Höfling, Suemi Marui, Carlos Alberto Buchpiguel, Giovanni Guido Cerri, Maria Cristina Chammas

**Affiliations:** ^1^Ultrasound Unit, Department of Radiology, University of São Paulo Medical School, Clinics Hospital, 05403-000 São Paulo, SP, Brazil; ^2^Thyroid Unit, Department of Endocrinology and Metabolism, University of São Paulo Medical School, Clinics Hospital, 05403-000 São Paulo, SP, Brazil; ^3^Radiology Institute (InRad), Department of Radiology, University of São Paulo Medical School, Clinics Hospital, 05403-000 São Paulo, SP, Brazil

## Abstract

**Background:**

The end-diastolic velocity (EDV) of thyroid arteries reflects peripheral blood flow resistance.

**Objective:**

The aim was to evaluate EDV correlations with other Doppler sonography parameters and with clinical and biochemical variables in a sample of patients with hypothyroidism caused by chronic autoimmune thyroiditis (CAT).

**Methods:**

A sample of 48 CAT hypothyroid patients receiving treatment with stable doses of levothyroxine was selected. The participants underwent clinical evaluation and measurement of serum thyrotropin (TSH), total triiodothyronine (T3), total thyroxine (T4), free T4, thyroid peroxidase antibodies (anti-TPO), and antithyroglobulin antibodies (anti-Tg) and Doppler sonography.

**Results:**

The EDV of the inferior thyroid arteries (ITA-EDV) was strongly and positively correlated with the peak systolic velocity of the inferior thyroid arteries (ITA-PSV, *r* = 0.919), thyroid volume (*r* = 0.711), and thyroid visual vascularization pattern (TVP, *r* = 0.687). There was no correlation between ITA-EDV and the clinical variables, hormones, anti-TPO, or anti-Tg.

**Conclusion:**

The strong correlation of ITA-EDV with ITA-PSV, TVP, and volume suggests that increased vascularization in CAT may be associated with a reduction in thyroid blood flow resistance, possibly due to an angiogenesis-induced increase in the total vascular cross-sectional area of the parenchyma.

## 1. Introduction

Doppler sonography is a noninvasive, widely available, reproducible diagnostic tool [1, 2] that has high sensitivity and specificity in the diagnosis and differentiation of autoimmune thyroid diseases [3–5].

In addition to providing B-mode sonography information, this technique allows glandular blood flow to be studied. Vascularization can be qualitatively investigated by estimating the visual thyroid vascularization pattern (TVP) or quantitatively investigated by measuring thyroid blood flow area. Doppler sonography also enables determination of peak systolic velocity (PSV) and end-diastolic velocity (EDV), as well as the resistive index (RI) and pulsatility indices of the superior, inferior, intrathyroid, and intranodular arteries [5, 6].

An initial report indicated that the diffusely increased TVP of the gland was pathognomonic of untreated Graves' disease (GD) [7]. A subsequent study demonstrated that patients with chronic autoimmune thyroiditis (CAT) may exhibit a TVP similar to that found in GD [8], making this parameter limited in its ability to differentiate between the 2 diseases. Evaluation using blood flow area or vascularization index is still rarely used in clinical practice due to the need for specific software. In CAT, TVP is often increased, both in patients with euthyroidism and in those with hypothyroidism [8].

The PSV of the superior thyroid arteries (STA-PSV), inferior thyroid arteries (ITA-PSV), and intrathyroid arteries has been investigated for both the diagnosis of autoimmune thyroid disease and differential diagnosis of CAT and GD patients [2, 4, 9]. Increased intrathyroid artery PSV was found in a study that included treated and untreated CAT hypothyroid patients.

Increased ITA-PSV was also observed in a sample of CAT euthyroid and hypothyroid patients compared to control subjects [4, 8]. When CAT and GD patients were compared, although the latter presented with higher EDV and PSV values, some of the values overlapped [4].

The underlying mechanisms that promote increased vascularization and blood flow velocity in the thyroid arteries in individuals with CAT are still poorly understood and are debated in the literature. Among the hypotheses proposed to explain this finding are autoimmune process activity, angiogenic factor production, and an increase in thyrotropin (TSH) [4, 6, 8, 10–12].

However, not all CAT patients have increased PSV and TVP, especially those with glandular atrophy [8, 10]. CAT patients with large goiters may have moderately increased PSV values, as well as a diffuse increase in TVP, which may be confused as patients with mild hyperthyroidism caused by GD and thus reduce the sensitivity and specificity of these parameters in the differential diagnosis of these diseases [4, 8]. Hence, finding factors that might affect gland vascularization is important.

Little is known about the possible associations between EDV and the various characteristics of CAT patients. Since EDV indirectly represents resistance to arterial blood flow [13], EDV could help to elucidate the reason for the vascular changes present in CAT and thus provide clues to improve the accuracy of Doppler sonography in these patients. The objective of this study was thus to evaluate the correlations between EDV and the various Doppler sonography parameters and clinical and biochemical variables in a homogeneous sample of patients with hypothyroidism caused by CAT who were being treated with levothyroxine.

## 2. Materials and Methods

### 2.1. Patients

This was a prospective study that included a sample of 48 patients with a previous diagnosis of hypothyroidism due to CAT at the Thyroid Outpatient Clinic of the Hospital das Clínicas, University of São Paulo Medical School (HC-FMUSP), where the population receives a sufficient intake of daily iodine [14]. Doppler sonography was conducted at the Radiology Institute of HC-FMUSP.

The Research Ethics Committee of HC-FMUSP approved this study and the patient consent forms. All the patients signed the consent forms voluntarily.

### 2.2. Sample Collection

The following patients were included: adult CAT hypothyroid patients who had been receiving treatment with stable doses of levothyroxine and had normal (or near normal) serum levels of total triiodothyronine (T3), total thyroxine (T4), free T4 (fT4), and TSH. The appropriate levothyroxine replacement dose for each patient was determined by a doctor of the Thyroid Outpatient Clinic. CAT was previously diagnosed for patients who met the following criteria: (1) high serum levels of thyroid peroxidase antibodies (anti-TPO) and/or antithyroglobulin antibodies (anti-Tg); (2) an ultrasound (US) pattern compatible with CAT, that is, parenchyma with diffusely reduced echogenicity [15] and a diffusely heterogeneous texture [16]. All selected patients had clinical and biochemical hypothyroidism prior to levothyroxine replacement.

The exclusion criteria were as follows: (1) other causes of thyroiditis; (2) a history of GD; (3) prior treatment with radioiodine; (4) use of medications that could interfere with thyroid function, thyroid hormone and TSH level measurement, and autoimmune status; (5) previous thyroid or neck surgery; (6) mediastinal goiter; and (7) pregnancy.

### 2.3. Doppler Sonography Study

Examinations were conducted by an experienced investigator (Chammas, MC). The procedure was performed with the patients in the supine position with a cushion under their shoulders with their neck hyperextended.

### 2.4. Equipment

A high-resolution IU22™ device (Philips Medical Systems®, Bothell, WA, USA) attached to a broadband linear probe (5–12 MHz) was employed for the B-mode and Doppler sonography thyroid parameter analyses.

### 2.5. Gray Scale (B-Mode) Sonography

The size, volume, shape, echogenicity, and echotexture of the gland, as well as the presence or absence of both thyroid nodules and level VI cervical lymph nodes, were examined. The echogenicity was subjectively analyzed by comparing the intensity of the echoes from the thyroid with those of the sternocleidomastoid and prethyroid muscles. The thyroid parenchyma echo intensity was divided into 2 categories: normal or reduced echogenicity. The echotexture was categorized as either diffusely homogeneous or heterogeneous. The maximum measurements (centimeters) were obtained for the longitudinal (*L*, length), anteroposterior (*AP*, thickness), and transversal (width) axes of both lobes and the isthmus. The *L* and *AP* axes were obtained from the longitudinal plane, whereas the transversal axis was from the transverse images. The volume (*V*) (cubic centimeters) of each lobe and the isthmus was calculated using an ellipsoid model with the following formula: *V* = *L* × *AP* × *T* × 0.528. The left and right lobe volumes, as well as total thyroid volume (including the isthmus), were used as representative variables. Reference values of 6–16 cm^3^ [17] were adopted for the thyroid volume. The Doppler sonography images were obtained using power and pulsed Doppler imaging.

### 2.6. Color Doppler

**Figure 1:**
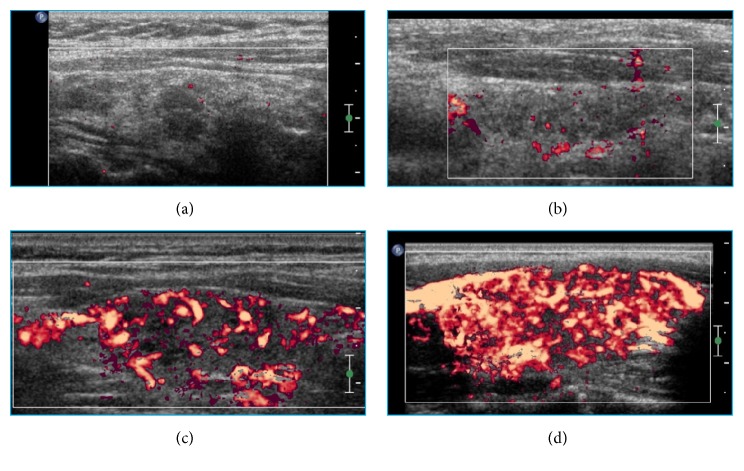
Color Doppler images. Parts (a), (b), (c), and (d) correspond to vascularization patterns 0, I, II, and III of the thyroid parenchyma, respectively.

To avoid underestimating vascularization intensity, the probe was lightly positioned on the skin without compression. The equipment was configured as follows: color Doppler gain around 80%; wall filter (WF) low, pulse-repetition frequency (PRF) of approximately 750 Hz; and velocity scale of 5.0 cm/s. The color gain was adjusted up to the highest possible level not associated with image saturation artifacts. All of the two-dimensional images were recorded at the time of greatest visible flow, corresponding to the PSV of blood flow. The TVP was classified into 4 categories, as previously planned (patterns 0, I, II, and III; Figure 1). Pattern 0: the vascularization is decreased and limited to the main peripheral arteries, which have reduced signals (Figure 1(a)). Pattern I: the vascularization is limited to the main peripheral thyroid arteries, which exhibit the usual signals, whereas only signals of vascularization focal points exist in the parenchyma with either a scattered distribution or a localized presence in the interiors of the nodules (Figure 1(b)). Pattern II: clearly increased vascularity is observed with a scattered distribution (Figure 1(c)). Pattern III: a marked increase in vascularization with a diffuse and homogeneous distribution is observed, including the so-called “thyroid inferno” pattern [18] (Figure 1(d)). We classified TVP separately for the right and left lobes of each patient's thyroid, thus obtaining a total of 96 lobes.

### 2.7. Pulsed Doppler

**Figure 2:**
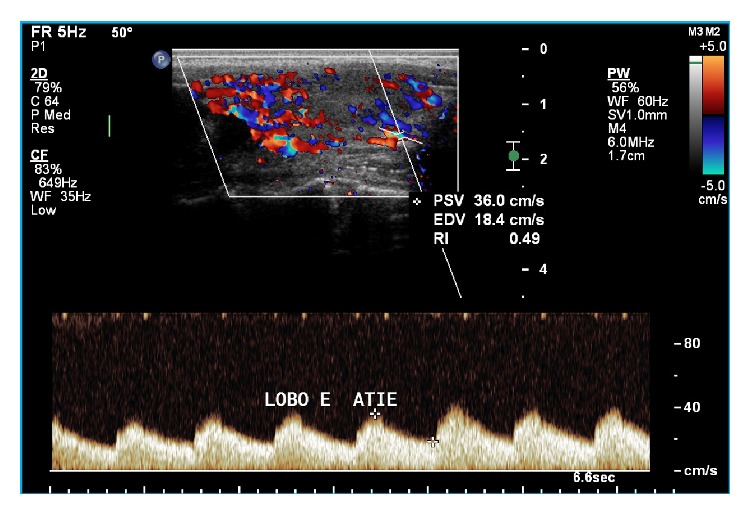
Evaluation of the PSV, EDV, and resistive index of the left inferior thyroid artery via pulsed Doppler analysis.

The PRF was set according to the speed of the flow and the parameters that yielded the best possible graphic representation. The parameters for pulsed Doppler included the following: Doppler frequency of 6.0 MHz; gain around 80%; and low WF. For PSV measurements, both the right and left inferior thyroid arteries (ITA) were identified, and the sampling volume was adjusted to 1 mm at the center of vessels. The Doppler angle was always corrected to values between 0° and 60°, so that it was parallel to the direction of blood flow. Both the right and left ITA were examined along the oblique axial plane, close to the transition between the middle and inferior third of the right and left thyroid lobes, respectively. To evaluate the ITA, the sample-volume cursor was positioned close to the trachea to avoid artifacts coming from the common carotid artery and the internal jugular vein. All spectral waveform images were stored digitally (Figure 2). The PSV, EDV, and RI values in the right and left ITA (right and left ITA-PSV, ITA-EDV, and ITA-RI, resp.) were obtained from pulsed Doppler analysis. The mean values of the right and left ITA-PSV, ITA-EDV, and ITA-RI were also calculated. The values found for the right and left ITA-PSV, ITA-EDV, and ITA-RI were compared to the respective right and left thyroid lobe volume and TVP data. The mean values obtained for the right and left ITA-PSV, ITA-EDV, and ITA-RI were compared to the total gland volume.

### 2.8. Biochemical Measurements

Venous blood samples were obtained by direct venous puncture in the morning after an overnight fast and before any medication was ingested. The serum levels of total T3, total T4, fT4, TSH, anti-TPO, and anti-Tg were measured through the chemiluminescence method using total T3, total T4, fT4, TSH, anti-TPO, and anti-Tg ADVIA Centaur® XP Immunoassay System kits (Siemens Healthcare Diagnostics Inc., Tarrytown, NY, USA) prior to the Doppler sonography examinations. All the biochemical exams were performed by the laboratory Alta Excelência Diagnóstica-DASA Group, São Paulo, SP, Brazil. The reference values, analytical sensitivities, intra-assay coefficients of variations, and interassay coefficients of variations for the aforementioned assays are as follows, respectively: (A) total T3 = 70–220 ng/mL, 20 ng/dL, 2.22%, and 1.15%; (B) total T4 = 5.1–13.5 *μ*g/dL, 0.3 *μ*g/dL, 2.04%, and 2.98%; (C) fT4 = 0.7–1.8 ng/dL, 0.1 ng/dL, 2.7%, and 2.94%; (D) TSH = 0.4–4.3 *μ*UI/mL (15 to 61 years of age) or 0.4–5.8 *μ*UI/mL (over 61 years of age), 0.008 *μ*UI/mL, 2.02, and 1.55%; (E) anti-TPO < 60 UI/mL, 1.0 UI/mL, 4.1%, and 3.1%; (F) anti-Tg < 60 UI/mL, 1.0 UI/mL, 4.3%, and 3.0%. When serum anti-TPO and anti-Tg levels were below or above the detection limit, the respective minimum and maximum values were adopted as representative for statistical analysis.

### 2.9. Statistical Analysis

The statistical analysis was conducted using SPSS® software (version 17.0–SPSS, Inc., IBM®, Chicago, IL, USA). Descriptive statistics were calculated for continuous variables, and the normality assumption was verified using the Kolmogorov-Smirnov test. The baseline clinical data are presented as the mean ± standard deviation (SD) for continuous variables and as the median (quartiles) for categorical variables. The correlation between variables was evaluated using the Pearson coefficient for variables with a normal distribution and the Spearman coefficient for variables that did not follow a normal distribution. Correlations between the following variables were tested: height, weight, body mass index (BMI), disease duration, daily levothyroxine replacement dose, serum levels of total T3, total T4, fT4, TSH, anti-TPO, anti-Tg, total thyroid gland volume, right lobe volume, left lobe volume, right lobe TVP, left lobe TVP, TVP of all lobes, right and left ITA-PSV values, mean right and left ITA-PSV values, right and left ITA-EDV values, mean right and left ITA-EDV values, RI of the right and left ITA, and mean RI of the right and left ITA. “Stepwise” multiple linear regression was applied for multivariate analysis. Doppler sonography parameters (thyroid volume, ITA-EDV, ITA-PSV, ITA-RI, and TVP) were considered dependent variables, whereas clinical and biochemical variables were considered independent variables when fitting multiple linear regression models with the stepwise procedure for variable selection. The estimated parameter, standard error, descriptive level of probability (*P*), and coefficient of determination (*R*^2^) are presented for each model. Two-sided *P* values < 0.05 were considered statistically significant ( ^*∗*^).

## 3. Results

### 3.1. Levothyroxine Dose and Biochemical Measurements of CAT Patients

The 48 patients included in the study were receiving stable doses of levothyroxine for replacement therapy and exhibited normal serum thyroid hormone levels and normal (or near normal) TSH levels. Six of the 48 patients had minor TSH changes but normal thyroid hormones. The TSH levels of 3 patients were below the reference value: 0.21 *μ*UI/mL, 0.27 *μ*UI/mL, and 0.33 *μ*UI/mL. The TSH levels of 3 other patients were above the reference value: 5.22 *μ*UI/mL, 5.34 *μ*UI/mL, and 6.28 *μ*UI/mL. The other participants presented with normal TSH and thyroid hormone values. All patients showed elevated levels of anti-TPO and/or anti-Tg. Forty-six of the 48 (95.83%) patients in the sample had high levels of anti-TPO, 32 (66.67%) had elevated anti-Tg, and 30 (62.50%) had an increase in both autoantibodies.

### 3.2. B-Mode Sonography Parameters

All patients (100%) exhibited parenchyma with diffusely reduced echogenicity and diffusely heterogeneous texture. Contour irregularity was identified in 32 (66.67%) patients; hyperechoic septa crossing the parenchyma compatible with fibrosis were found in 26 (54.17%) patients; and hyperechogenic (dense) lines and points dispersed throughout the parenchyma, compatible with fibrosis and/or calcifications, were found in 7 (14.58%) patients. In 39 (81.25%) patients, hypoechogenic areas with poorly defined margins were found in the parenchyma, which may be associated with lymphocytic infiltrate. Hyperechogenic areas were found in the thyroid parenchyma of 8 (16.67%) patients. Nodules were found in 7 (14.58%) patients. Level VI reactive (inflammatory) lymph nodes were identified in all evaluated patients (100%).  Table 1 shows the results of descriptive statistical analysis of all study variables, except for TVP, which is shown separately. Of the 48 patients, 30 (62.50%) had a normal thyroid volume, and 9 (18.75%) had increased volume (goiter), while 9 (18.75%) had reduced volume (atrophic thyroiditis). Of the 30 patients with a normal volume, 27 (90.00%) had increased TVP, while only 1 (3.33%) had reduced TVP, and 2 had normal TVP (6.66%). Among the 9 patients with increased volume, only 1 (11.11%) had reduced TVP, while the other 8 patients (88.89%) had increased TVP. Of the 9 patients with decreased volume, 5 showed a decrease in TVP (55.55%), while 2 had increased TVP (22.22%), and 2 had a normal TVP (22.22%). The right lobe TVP classification was the same as that of the left lobe in all evaluated patients.  Table 2 shows the distribution of TVP categories for the right and left thyroid lobes. TVP was increased in 72 of the 96 evaluated lobes (75.00%), reduced in 14 (14.58%), and normal in 10 (10.42%).

### 3.3. Correlations between All Variables: Doppler Sonography and Clinical and Biochemical Data

**Figure 3:**
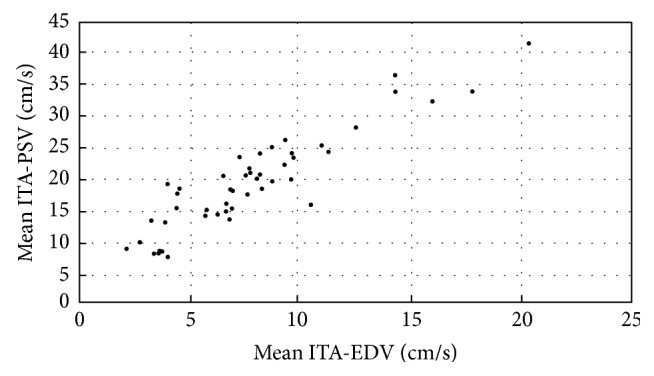
Scatterplot showing the relationship between the mean ITA-EDV (cm/s) and mean ITA-PSV (cm/s): *r* = 0.919; *P* < 0.001.

A strong correlation was found between the right and left ITA-EDV and the respective ITA-PSV (*r* = 0.880, *P* < 0.001; *r* = 0.920, *P* < 0.001). The mean value obtained from the right and left ITA-EDV was also strongly correlated (Figure 3) with the mean right and left ITA-PSV (*r* = 0.919; *P* < 0.001).

There was a weak but significant negative correlation between the right and left ITA-EDV and the respective right and left ITA-RI (*r* = −0.495, *P* < 0.001; *r* = −0.485, *P* < 0.001). The mean right and left ITA-EDV also showed a weak negative correlation with the mean right and left ITA-RI (*r* = −0.494; *P* < 0.001).

**Figure 4:**
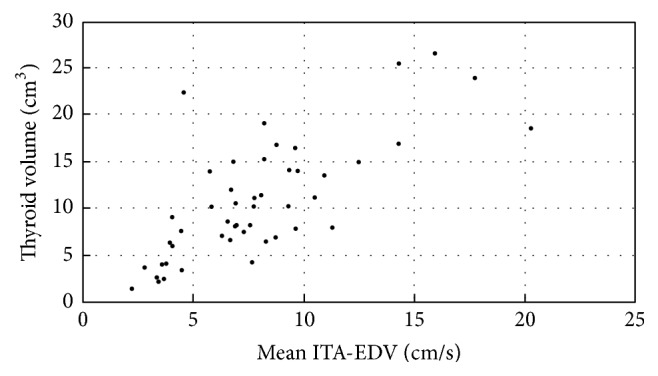
Scatterplot showing the relationship between the mean ITA-EDV (cm/s) and thyroid volume (cm^3^): *r* = 0.711; *P* < 0.001.

The right and left ITA-EDV correlated with the right and left lobe volumes, respectively (*r* = 0.623, *P* < 0.001; *r* = 0.599, *P* < 0.001). The total thyroid volume exhibited a strong correlation with the mean right and left ITA-EDV (*r* = 0.711; *P* < 0.001; Figure 4). There was no correlation between ITA-EDV and clinical and biochemical parameters.

**Figure 5:**
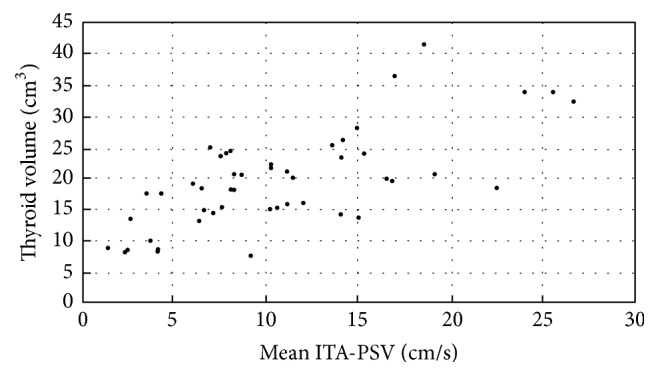
Scatterplot showing the relationship between the mean ITA-PSV (cm/s) and thyroid volume (cm^3^): *r* = 0.700; *P* < 0.001.

The right and left ITA-PSV values correlated with the right and left thyroid lobe volumes, respectively (*r* = 0.642, *P* < 0.001; *r* = 0.590, *P* < 0.001). The mean right and left ITA-PSV (Figure 5) correlated strongly with the total gland volume (*r* = 0.700; *P* < 0.001). ITA-PSV and ITA-RI were not correlated with clinical and biochemical parameters.

TVP was correlated with the mean right and left ITA-EDV (*r* = 0.687; *P* < 0.001) and the mean right and left ITA-PSV (*r* = 0.681; *P* < 0.001). TVP was correlated with the total gland volume (*r* = 0.519; *P* < 0.001) but not with the mean right and left ITA-RI (*r* = −0.280; *P* = 0.053). There was a weak negative correlation of TVP with fT4 levels (*r* = −0.333; *P* = 0.024). TVP did not correlate with the anti-TPO and anti-Tg levels.

The mean right and left ITA-RI showed a negative correlation with the total thyroid volume (*r* = −0.337; *P* = 0.019). The left ITA-RI had a poor negative correlation with the left lobe volume (*r* = 0.333; *P* = 0.021), but no correlation was found between the right ITA-RI and right lobe volume (*r* = −0.282; *P* = 0.052).

### 3.4. Association between Doppler Sonography Variables and Clinical and Biochemical Parameters

A stepwise linear regression analysis was performed to define the relative contribution of different parameters (independent variables) to thyroid ITA-EDV, ITA-PSV, TVP, and thyroid volume (dependent variables). ITA-EDV and ITA-PSV were not related to any of the clinical and biochemical variables. Increased patient height was related to increased thyroid volume (Table 3). An increase in anti-Tg and fT4 levels was related to a reduction in the TVP in the thyroid lobes (Table 3). An increase in the age and anti-Tg level variables led to an increase in the mean ITA-RI values, whereas the height and anti-TPO levels were related to a reduction in the mean ITA-RI (Table 3).

## 4. Discussion

One advantage of this study was that a homogeneous sample could be selected, which consisted only of CAT patients with established hypothyroidism under treatment with stable and sufficient doses of levothyroxine to maintain normal T3, T4, and fT4 levels and normal (or near normal) TSH. This homogeneity excluded the possibility that the thyroid hormone and TSH levels could have significantly affected the parameters analyzed in Doppler sonography.

The results of this study demonstrate that ITA-EDV is strongly correlated with ITA-PSV and with thyroid volume in CAT patients. In addition, TVP also correlated with ITA-EDV, ITA-PSV, and gland volume. These findings may help to explain why some CAT patients also have increased PSV [4, 6, 8] and TVP, as occurs in GD [4, 10, 18].

Elevated ITA-PSV was reported as the Doppler sonography parameter with the highest sensitivity and specificity for differential diagnosis among thyroid autoimmune diseases [4, 10, 18]. However, for some CAT and GD patients, both PSV and TVP may present false-positive and false-negative results, which reduce the accuracy of Doppler sonography in the differential diagnosis of these diseases [4, 19].

Previous studies have shown that both TVP [6, 8, 20] and ITA-PSV are increased in patients with CAT [6, 18] compared with those in control subjects. Although hypotheses have been proposed to explain these alterations in CAT, the underlying mechanisms are not yet fully understood. Among them are the stimulation of TSH and the autoimmune process activity [4, 8, 10, 12, 20]. The stimulation of angiogenesis (i.e., the sprouting of new blood vessels from preexisting capillaries) [10, 11, 21] might also be a possible cause of increased TVP and PSV.

EDV is considered a Doppler parameter that reflects peripheral resistance [13, 22, 23]. Increased blood flow resistance in the carotid and cerebral arteries have caused a reduction in EDV [24]. EDV has been poorly evaluated in CAT patients, especially when considering the associations with clinical, biochemical, and Doppler sonography variables. EDV is higher in CAT patients than in individuals without thyroid disease [4]. This increase, in theory, reflects a reduction in the arterial blood flow resistance of the thyroid. A key study, published by Tseleni-Balafouta et al. [25], morphologically documented the presence of angiogenesis in thyroid parenchyma of CAT patients, demonstrating a significant increase in microvessel density in histologic sections. In addition, the authors reported intrathyroid vessel fusion in CAT [25]. The addition of more blood vessels to a parallel vascular circuit, such as the thyroid, reduces vascular resistance [26] and consequently increases blood flow. However, the velocity (*v*) of blood flow (*F*) is inversely proportional to the vascular cross-sectional area (*A*): *v* = *F*/*A* [26]. With this reasoning, the increase in *A* of the parenchyma most likely leads to reduced flow velocity in the intrathyroid vessels. In the thyroid arteries, however, it is possible that the opposite occurs because the increased cross-sectional area of these vessels is limited to their vasodilation. Thus, there would be an increase in flow without a proportional increase in area, which would lead to increased blood flow velocity in the thyroid arteries. It is also possible that thyroid artery vasoconstriction occurs to promote blood flow control according to the metabolic needs of the gland, a mechanism that would reduce *A* of the thyroid arteries and contribute to an increase in *v*. In fact, high velocity flow in diastole has been verified in the presence of a low resistance flow pattern [27]. The correlations between TVP and both ITA-EDV and ITA-PSV observed by our study group reinforce the relationship between *A* and *v*.

A reduction in arterial resistance in the thyroid parenchyma may, therefore, also be the cause of increased PSV. The strong correlation between ITA-EDV and ITA-PSV found in this study corroborates this hypothesis and would explain why the ITA-EDV and ITA-PSV values of some CAT patients are higher than those with a normal thyroid [4].

Correlations between thyroid volume and both ITA-EDV and ITA-PSV were demonstrated. The increase in volume might be due to the presence of intense lymphocytic infiltrate, as well as a pronounced stimulus of angiogenesis, which would increase both EDV and PSV. The correlations between CXCL-10, a chemokine that plays an important role in modulating angiogenesis, and ITA-PSV and thyroid volume found by others support this possibility [10].

The negative correlations between RI and both ITA-EDV and TVP suggest that an increase in the number of parenchymal blood vessels (↑ TVP) induces a reduction in vascular resistance (↓ RI) and elevation in ITA-EDV. However, no correlation was found between RI and ITA-PSV. This result might be explained by the fact that RI is affected not only by vascular resistance but also by vascular compliance [28, 29].

An association between both ITA-EDV and ITA-PSV and both anti-TPO and anti-Tg levels was not found in our study. Previous studies have shown a correlation between TVP and/or PSV and anti-TPO and anti-Tg serum levels [8, 20]. However, RI was associated with anti-TPO and anti-Tg levels in our sample. These results suggest that, in addition to autoantibodies, other factors certainly contribute to autoimmune process activities, such as immune system cells, cytokines, and chemokines.

Most patients with CAT exhibited TVP II and III. This result corroborates those of Schulz et al. [8], showing that increased TVP is frequently observed among patients with CAT. Most patients with goiters had increased TVP, whereas the majority of those with atrophic thyroiditis exhibited decreased TVP. The correlation between volume and TVP suggests that the increase in the number of intrathyroid vessels contributes to increase of the volume as well as corroborating previous results [10].

The increase in TVP and PSV in CAT hypothyroid patients has been suggested to be dependent on the TSH stimulus [12]. In our study, the majority (75.5%) of the analyzed thyroid lobes showed increased TVP, whereas the TSH concentrations were normal (or near normal). This finding clearly demonstrates that TVP may be increased even at normal TSH levels. Thus, in addition to TSH, other mediators of the immune response must contribute to the induction of vascular changes in CAT.

In our opinion, thyroid volume might indirectly represent a histological change in the glandular parenchyma associated with CAT. Larger glands might have more intense autoimmune inflammatory processes marked by diffuse lymphocytic infiltration and intense angiogenesis. An increase in the number of blood vessels would result in an increase in the total *A* of the gland, which would lead to a decrease in blood flow resistance and thus explain the elevations in ITA-EDV and ITA-PSV found in this study. In atrophic thyroiditis, however, the parenchyma is predominantly replaced by fibrosis [30], which leads to a reduction in *A* and, consequently, to an increase in blood flow resistance and the reduction of ITA-EDV and ITA-PSV.

Due to its wide availability, Doppler sonography may be the first examination performed to differentiate between autoimmune thyroid diseases. In this case, it is important to remember that CAT patients, especially those with large goiters, may have moderately increased ITA-EDV and ITA-PVS values, which may overlap with the values of those who have mild forms of hyperthyroidism caused by GD. In these situations, the analysis of other Doppler sonography parameters such as the presence of a marked reduction in echogenicity, hyperechoic septa crossing the parenchyma, and level VI cervical lymph nodes with reactive appearance may define a CAT diagnosis [15, 31]. In our sample, all patients had this last finding.

One limitation of this study was that we analyzed a relatively small sample. Therefore, further research including more patients will be needed to confirm these results.

## 5. Conclusion

The results showed strong correlations among ITA-EDV, ITA-PSV, TVP, and thyroid volume. The relationship between these variables indicates that the vascularization changes observed in CAT patients may be caused by a variation in thyroid blood flow resistance due to an increase in the number of blood vessels caused by angiogenesis. Future studies will be important, not only to confirm these findings but also to elucidate the mechanisms that promote angiogenesis in this disease.

## Figures and Tables

**Table 1:** Clinical, biochemical, and sonographic characteristics of the CAT patients.

Variable	Mean	SD	Minimum data value	Maximum data value
Age (years)	45.17	11.50	20.00	68.00
Female/male	47/1			
Height (m)	1.58	0.06	1.48	1.81
Weight (kg)	72.57	16.66	46.10	120.00
BMI (kg/m^2^)	29.19	6.42	18.12	46.88
Disease duration (years)	11.92	3.36	7.00	23.00
Levothyroxine dose (*μ*g/day)	112.55	46.67	0.00	275.00
Total T3 levels (ng/mL)	102.34	18.05	61.00	137.00
Total T4 levels (*μ*g/dL)	10.00	1.98	6.20	13.80
Free T4 levels (ng/dL)	1.25	0.28	0.73	1.77
TSH levels (*μ*UI/mL)	2.06	1.51	0.21	6.28
Anti-TPO levels (U/mL)	621.11	0.28	13.10	3,000.00
Anti-Tg levels (U/mL)	399.36	803.25	20.40	4,000.00
Thyroid volume (cm^3^)	10.74	6.21	1.42	26.60
Right lobe volume (cm^3^)	5.43	3.25	0.90	14.20
Left lobe volume (cm^3^)	4.41	2.88	0.47	12.70
Right ITA-PSV (cm/s)	19.97	8.47	6.33	51.50
Left ITA-PSV (cm/s)	19.11	8.99	5.84	40.80
Mean ITA-PSV (cm/s)	19.54	7.67	7.62	41.50
Right ATI-EDV (cm/s)	7.93	4.31	2.48	26.10
Left ITA-EDV (cm/s)	7.65	4.20	1.92	18.50
Mean ITA-EDV (cm/s)	7.86	3.90	2.20	20.30
Right ITA-RI	0.62	0.08	0.45	0.80
Left ITA-RI	0.61	0.08	0.45	0.79
Mean ITA-RI	0.61	0.08	0.47	0.80

SD = standard deviation.

**Table 2:** Distribution of TVP categories in the right and left thyroid lobes.

TVP	Right		Left	
	*N*	%	*N*	%
0	7	14.58	7	14.58
I	5	10.42	5	10.42
II	19	39.58	19	39.58
III	17	35.42	17	35.42

Total	48	100.00	48	100.00

**Table 3:** Stepwise linear regression analysis results.

Dependent variables	Independent variables	Estimate parameter	Standard error	*P*	*R* ^2^
Thyroid volume (cm^3^)	(Constant)	−39.30565	22.63403	0.089	0.100
	Height	31.79383	14.34889	0.032	

TVP of all lobes	(Constant)	3.47801	0.63938	0.000	0.200
	Anti-Tg levels	−0.00047	0.00017	0.009	
	Free T4	−1.08421	0.50227	0.037	

Mean ITA-RI	(Constant)	1.24694	0.25758	<0.001	0.438
	Age	0.00178	0.00082	0.034	
	Anti-Tg levels	0.00003	0.00001	0.006	
	Height	−0.44762	0.15376	0.006	
	Anti-TPO levels	−0.00003	0.00001	0.025	
